# Diversity and use of medicinal plants for soup making in traditional diets of the Hakka in West Fujian, China

**DOI:** 10.1186/s13002-019-0335-y

**Published:** 2019-11-28

**Authors:** Binsheng Luo, Feifei Li, Selena Ahmed, Chunlin Long

**Affiliations:** 1 College of Life and Environmental Sciences, Minzu University of China, Beijing, 100081 China; 2 Key Laboratory of Ethnomedicine (Minzu University of China), Ministry of Education, Beijing, 100081 China; 3 State Key Laboratory of Environmental Criteria and Risk Assessment, Chinese Research Academy of Environmental Sciences, Beijing, 100012 China; 4 The Food and Health Lab, Department of Health and Human Development, Montana State University, Bozeman, MT 59717 USA; 5 Kunming Institute of Botany, Chinese Academy of Sciences, Kunming, 650201 China

**Keywords:** Traditional food systems, Medicinal plants, Edible plants, Ethnonutrition, Traditional ecological knowledge

## Abstract

**Background:**

Wild edible and medicinal plants were an important component of traditional diets and continue to contribute to food security, nutrition, and health in many communities globally. For example, the preparation and consumption of soup made of medicinal plants for promoting health and preventing disease are a key component of the traditional diets of the Hakka socio-linguistic group of China’s West Fujian Province. As environmental and socio-economic factors drive the shift away from traditional diets, there is a need for ethnobotanical documentation of the diversity of wild edible and medicinal plants as well as associated knowledge and practices.

**Method:**

Ethnobotanical surveys were conducted in Hakka communities in West Fujian Province between 2017 and 2018 to document plants used in medicinal soups as well as associated traditional ecological knowledge, practices, and conservation status. Surveys included semi-structural interviews, key informant interviews, participatory rural appraisal, and focus group discussions. Quantitative indices, including cultural food significance index (CFSI) and relative frequency of citation (RFC), were calculated to evaluate the importance of documented plants to Hakka communities. The species with the highest CFSI and RFC values were ranked by informants and further evaluated according to their individual properties and growth environment.

**Results:**

A total of 42 medicinal plant species, belonging to 25 families and 41 genera, were documented for making soup by the Hakka. The Asteraceae botanical family was the most prevalent, and their root or the entire plant is used for soup making. Informants incorporate different ingredients in soups for their flavors as well as medicinal properties on the basis of the local ethnonutrition system. The most prevalent medicinal uses of the documented plants for making soups were used for clearing inner *heat* (58.1% of the species), treating inflammation (37.2%), and counteracting *cold* in the body (20.9%). Informants perceived that the medicinal properties of soup-making plants are influenced by the time of harvest, the local environment, and the climate.

**Conclusion:**

Efforts are needed to preserve the ecological knowledge associated with traditional diets towards supporting both environmental and human well-being in rapidly developing communities experiencing the nutrition transition and biodiversity loss.

## Background

A primary challenge of our time is supporting food security and public health while conserving ecological resources in socially acceptable ways [1]. However, poor diets are the leading risk factor of disease globally with malnutrition impacting every nation, including undernutrition, micronutrient deficiencies, excess weight, obesity, and diet-related non-communicable diseases [2]. At the same time, food production is recognized to place a greater burden on ecosystems compared with other human activities [3]. In response to these challenges, efforts to promote healthy and sustainable food systems are called for that support both environmental and human well-being [4]. Previous studies have supported that traditional food systems of communities with an intimate understanding of their surroundings can provide strategies for reconciling ecological well-being and food production [5].

Traditional food systems are place-based food systems where foods are procured from the surrounding natural environment [6] and are part of a cultural heritage where food is entwined with identity and health of local communities [7]. Wild edible and medicinal plants were primary components of traditional food systems and continue to contribute to food security, nutrition, and health in many communities globally [5]. For example, the preparation and consumption of soup made of medicinal plants for promoting health and preventing disease are a crucial component of traditional food systems and ethnonutrition perspectives of the Hakka socio-linguistic group of China’s West Fujian Province. Such traditional food systems promote sustainability on the basis of environmental, health, cultural, and economic dimensions. For instance, traditional food systems support the economic dimension of sustainability by providing a non-market source of diverse foods without a financial cost [8]. Also, traditional food systems support the environmental dimension of sustainability by encouraging continued use and sustainable harvest of wild foods, thus encouraging environmental stewardship [9].

However, numerous environmental and socio-economic factors have led to changes in food systems around the world [10]. These factors include economic growth, globalization, trade liberalization, urbanization, industrialization, technological changes, mass media growth, depletion of natural resources, and climate change [10]. Shifts in these environmental and socio-economic factors have coincided with a sequence of changes in food procurement strategies and diets, as well as associated nutritional and health outcomes in what has come to be termed the nutrition transition [11]. The nutrition transition depicts dietary patterns shifting from traditional diets towards diets high in saturated fat, sugar, highly processed foods, and meat while being low in fiber and fruits and vegetables [11]. These changes in dietary patterns are reflected in shifts in nutritional and health outcomes, including increased weight status and diet-related non-communicable chronic disease [11, 12].

As environmental and socio-economic factors drive the shift away from traditional diets, there is a need for ethnobotanical documentation of the diversity of wild edible and medicinal plants as well as associated knowledge and practices of traditional food systems. It is well known that plentiful plants can be both used as food and medicine since ancient times; many cultures even do not make a strong distinction between food and medicine [13, 14]. This phenomenon of dietary therapy is also common in China and is consistent with traditional Chinese medicine [15]. The plant using as both medicine and food, usually possess lower safety risk to the human body and higher exploring potential which may help to improve our modern food system and develop new dietary supplement [13]. This paper seeks to address this need through ethnobotanical surveys in Hakka communities in China’s West Fujian on traditional food systems. Specifically, we focused on plants used in medicinal soups as well as associated traditional ecological knowledge, practices, and conservation status.

The Hakka are an ancient subgroup of the Han socio-linguistic group that maintain a unique way of life in China, including a traditional food system and active awareness of their nutrition and health [16]. There are approximately 50 million Hakka people distributed throughout China, particularly in the mountainous terrain of southeast China [17]. The Hakka migrated from the ancient Zhongyuan Region (currently Henan Province) to the southeast region of China in 300 AD to avoid wars and natural disasters [18]. Therefore, “Hakka” in Chinese Mandarin is called as “Kejia,” which means guests. It is considered that the mountainous terrain of the Hakka territory provides a natural barrier for protection from disputes with other groups and natural disasters while fostering the development of a distinct culture [18, 19]. Due to the long-term adaptation to the natural environment and close interactions with each other in isolated mountainous conditions, the Hakka developed a unique understanding of their surroundings, including the use of plants for medicinal purposes [19, 20].

The Hakka people are well-known for their longevity due to their healthy lifestyle, which includes a diet based on traditional food habits, including prevalent consumption of local fresh whole foods such as fruits, vegetables, fish, rice, and soybeans [21, 22]. In general, the Hakka use cooking methods that retain the natural flavor of the food [23]. They commonly use medicinal plants as crucial ingredients in their cuisine [24]. The Hakka also devote particular care to how different foods are paired together, as their ethnonutrition and culinary systems are based on the belief that different ingredients possess complementary properties that can work in synergy to improve human health [19].

Medicinal soups are a dietary staple of the Hakka traditional food system that is incorporated into every meal [8] towards improving the physical condition and long-term health [9]. According to the Hakka ethnonutrition system, the consumption of soups prepared with medicinal plants serves to facilitate digestion while providing additional nutrients and medicinal benefits [25]. The harvesting of edible plants from local community surroundings for the preparation of medicinal soups reflects the traditional ecological knowledge of the Hakka in using their natural resources to support well-being and prevent disease.

The traditional food systems of the Hakka are associated with healthy nutritional patterns and health outcomes, including balanced sodium to potassium ratio, low hypertension, and low mortality from cardiovascular diseases [16]. With an increasing emphasis on healthy diets for supporting health in a world that is progressively experiencing the burdens of diet-related chronic disease, it is imperative that traditional food systems such as that of the Hakka being documented. There has been limited documentation of Hakka traditional food systems in the literature, with one of our previous studies on Cantonese *slow*-cooked soup partially addressing Hakka cuisine [25]. Most studies involving ethnobotany of the Hakka have focused on the use of medicinal plants and cooling herbal tisanes [19, 23, 25, 26]. In this study, we investigated the ethnonutrition practice of soup-making in Hakka communities, including how traditional knowledge of medicinal plants and health practices is incorporated into current diets. Ethnobotanical findings from this study can be applied to inform the development of conservation, food, and nutrition programs that support both environmental and human well-being.

## Methods

### Study area

**Fig. 1:**
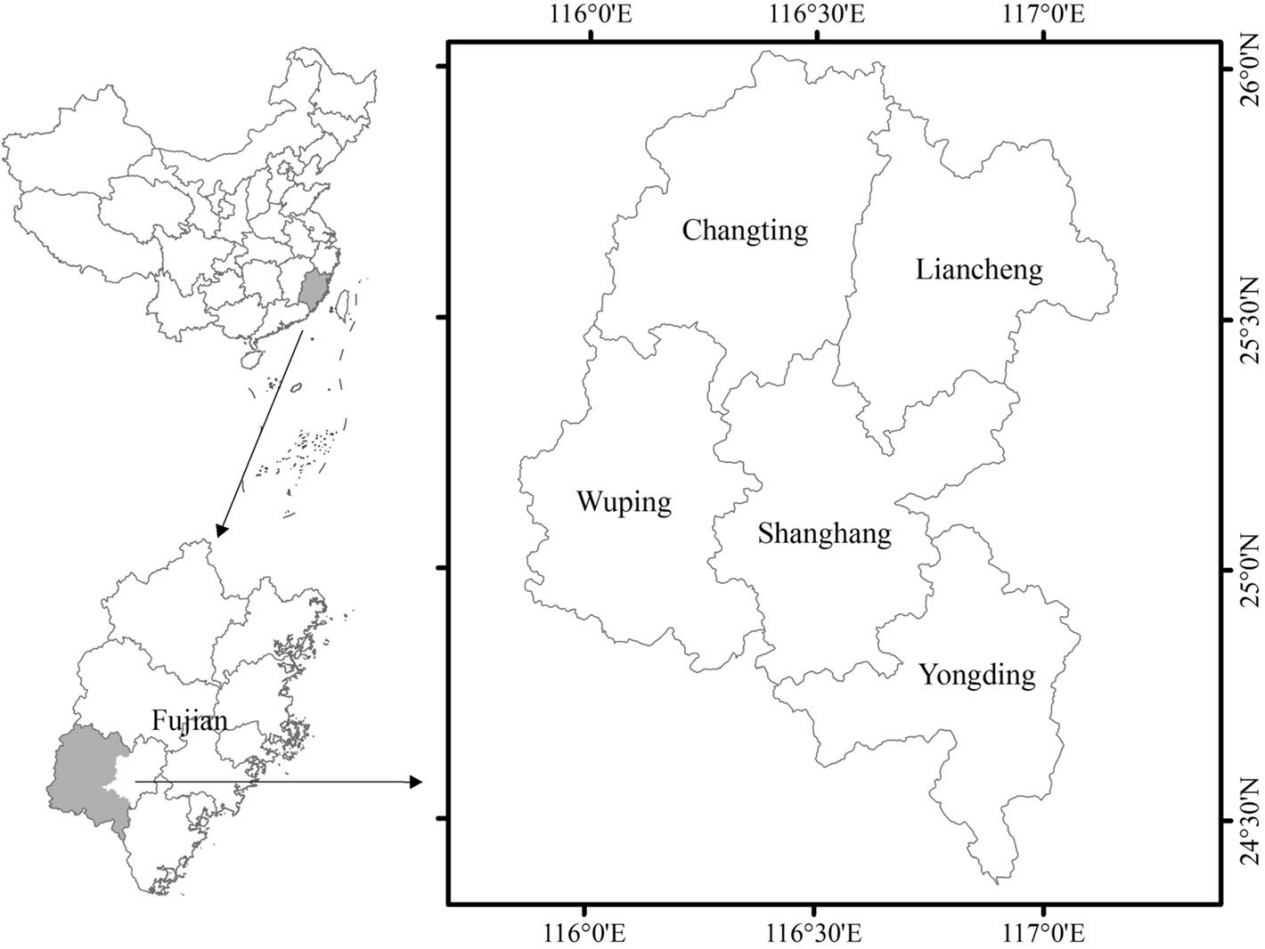
Study area in West Fujian Province in China

The “Hakka homeland” and center of population is in West Fujian, which adjoins East and North Guangdong and South Jiangxi, and encompasses Longyan City and Sanming City [27]. The study area locates in Longyan City, and the warm and humid subtropical monsoon climate there is characterized by rich biodiversity, including medicinal plants. According to the government statistics of Longyan City, there are about 78% of the land covered by forests, and around 75% population are Hakka people [28, 29]. This research was carried out in Changting, Yongding, Shanghang, Wuping, and Liancheng counties; these counties all belong to Longyan City and were selected because they possess well-defined Hakka characteristics with regard to a traditional economy, lineage composition, religion, and food culture (Fig. 1).

### Literature review

Prior to conducting the fieldwork, a literature review was carried out on Hakka culture and traditional food systems to develop an integrated knowledge framework for informing the design of survey instruments [17–19, 21, 23, 25–27]. This framework was developed based on the literature review, preliminary field research in Hakka communities, and ethnobotanical methods. The literature review involved searching multiple scientific databases and more general knowledge websites to learn about Hakka culture, including language, living habitat, and migration history. Additionally, anecdotal evidence of the Hakka diet and medicinal use of plants was obtained from local and regional publications of cities and counties in West Fujian Province.

### Ethnobotanical surveys

Ethnobotanical field surveys were carried out from 2017 to 2018 in the Hakka-dominant areas of West Fujian, including Changting, Yongding, Shanghang, Wuping, and Liancheng counties. As the first author of the paper is a native Hakka in the study area and speaks the local language, the communication with local Hakka participants was fluent and efficient. A total of 160 informants were interviewed, including herb vendors, buyers, folk healers, and knowledgeable elders. After obtaining informed consent from participants [30] based on ethical guidelines for the incorporation of human subjects in research, we carried out semi-structured interviews aiming to collect information about soup-making plants and associated traditional knowledge of local Hakka people. The questions basically included the following: (1) What plant and what part do you use for making soup; (2) Why do you use this species; (3) How do you process it; (4) Where and when do you collect it; (5) How often do you consume this species. In addition, participatory rural appraisal (PRA), key informant interviews, and focal group discussions were carried out to supplement the semi-structured interviews of the research.

Voucher specimens of plants used in medicinal soups were collected with the facilitation of local Hakka residents and identified by referencing *Flora of China* and Chinese floristic databases. All specimens were deposited into the Herbarium of the College of Life and Environmental Sciences at the Minzu University of China. An inventory of the local plants used in medicinal soups was made, which included vernaculars, scientific names, taxonomic status, life forms, parts used, times of harvest, ingredient combinations, and medicinal purposes (Table 1).

**Table 1:** Inventory of plants used to make medicinal soups by the Hakka people in West Fujian, China

Scientific name	Family name	Local name	Plant type	Part used	Time of Harvest	Match	Medicinal Properties	Specimen number
*Houttuynia cordata* Thunb.	Saururaceae	[ɡie^53^tiŋ^53^tsɿ^53^]	Herb	Whole plant	Summer	Crucian	Clear *heat*, induce diuresis, boost immunity	HKSP-02
*Litsea cubeba* (Lour.) Pers.	Lauraceae	[tsʰuŋ^55^tsɿ^53^fo^55^ɡæn^55^]	Shrub	Root	Whole year	Chicken, pig feet	Treat puerperal fever, treat rheumatism, relieve swelling and pain	HKSP-40
*Smilax glabra* Roxb.	Smilacaceae	[ŋɔn^55^pʰoŋ^53^tʰie^11^]	Shrub	Rhizome	Summer and fall	Pork ribs	Detoxify, treat rheumatism	HKSP-36
*Anoectochilus roxburghii* (Wall.) Lindl.	Orchidaceae	[tɕaŋ^55^ɕin^241^liŋ^11^]	Herb	Whole plant	Summer and fall	Chicken, pig heart	Clear *heat*, treat cancer, treat bronchitis, reduce blood pressure	HKSP-18
*Pholidota chinensis* Lindl.	Orchidaceae	[soɡ^24^kɔŋ^55^lan^11^]	Herb	Whole plant	Fall	Pig bone, duck	Clear *heat*, nourish the stomachache, nourish yin, eliminate phlegm and stop cough	HKSP-16
*Hemerocallis citrina* Baroni	Xanthorrhoeaceae	[tɕaŋ^55^tsaŋ^55^tsʰu^241^]	Herb	Flower	June to September	Pork	Detoxify, induce diuresis, diminish inflammation, calm the nerves	HKSP-19
*Juncus effusus* L.	Juncaceae	[tæŋ^55^sɑŋ^55^tsʰau^53^]	Herb	Stem pith	Summer and fall	Pig heart	Clear* heat*, induce diuresis	HKSP-17
*Nelumbo nucifera* Gaertn.	Nelumbonaceae	[liŋ^11^tsɿ^53^]	Herb	seed	Summer	pork ribs, duck	Clear *heat*, nourish yin	HKSP-07
*Abrus cantoniensis* Hance	Fabaceae	[ɡai^55^ku^53^tsʰau^53^]	Shrub	Whole plant	Whole year	Pork, pork ribs, pigs feet	Clear* heat*, detoxify, treat hepatitis	HKSP-31
*Pueraria lobata* (Willd.) Ohwi	Fabaceae	[ku^53^]	Liana	Root	Fall and winter	Pork ribs, duck	Treat fever, prevent alcoholism, resolve phlegm to relive cough	HKSP-28
*Tadehagi triquetrum* (L.) H. Ohashi	Fabaceae	[soɡ^24^kiɛ^53^sæb^24^]	Shrub	Leaf and root	Summer and fall	Pork	Improve digestion, clear *heat*, induce diuresis	HKSP-38
*Uraria crinita* (L.) DC.	Fabaceae	[lau^24^vu^53^len^11^]	Shrub	Whole plant	Whole year	Pork, young chicken	Clear *heat*, improve digestion	HKSP-34
*Phymatopteris hastata* (Thunb.) Pic. Serm.	Polypodiaceae	[aɡ^53^tʃuo^53^tsʰau^53^]	Herb	Whole plant	Summer and fall	Pork	Clear *heat*, induce diuresis	HKSP-01
*Polygala fallax* Hemsl.	Polygalaceae	[kuŋ^55^iɑŋ^55^tsʰu^241^]	Shrub	Root	Summer and fall	Chicken, pork	Nourish, clear damp, postpartum recover, nourish the kidney and liver	HKSP-33
*Rosa laevigata* Michx.	Rosaceae	[tʰuŋ^11^kuŋ^24^tsɿ^53^]	Shrub	Fruit, root	October to November	Pig kidney	Tonify kidney, tonify Yang	HKSP-39
*Berchemia lineata* (L.) DC.	Rhamnaceae	[lo^24^tsʰea^53^xen^53^]	Shrub	Root	Fall and winter	Pig bone	Detoxify, diminish inflammation, treat rheumatism, treat hepatitis, treat hernia	HKSP-35
*Ficus gasparriniana* Miq. var. *laceratifola* (Levl. et Vant.) Corner	Moraceae	[pʰoɡ^24^fo^55^ŋie^11^nen^241^ɡæn^55^]	Shrub	Root	Whole year	Pig bone, chicken	Clear* heat*, nourish yin, improve digestion, treat gynopathy	HKSP-37
*Ficus hirta* Vahl	Moraceae	[eŋ^55^tsɿ^53^mau^11^tʰau^11^]	Shrub	Root	Whole year	Chicken	Improve eyesight, nourish yin, improve digestion, dispel dampness	HKSP-30
*Morus alba* L.	Moraceae	[suŋ^55^]	Tree	Root	Whole year	Pork	Clear *heat*, treat rheumatism	HKSP-42
*Gynostemma pentaphyllum* (Thunb.) Makino	Cucurbitaceae	[eŋ^53^nit^24^samŋ^11^]	Shrub	Stem and leaf	Summer and fall	Chicken	Diminish inflammation, resolve phlegm to relive cough, treat diabetes, depress blood pressure	HKSP-29
*Ricinus communis* L.	Euphorbiaceae	[pi^24^mo^11^ɡɔŋ^53^]	Herb	Stem	The Winter Solstice	Female duck	Clear *heat*, clear damp, nourish yin	HKSP-13
*Rhodomyrtus tomentosa* (Ait.) Hassk.	Myrtaceae	[tuŋ^24^liŋ^11^tsɿ^53^]	Shrub	Root	Whole year	Pigs feet	Treat chronic diarrhea, treat rheumatism, treat hepatitis, reduce blood fat	HKSP-41
*Osbeckia chinensis* L.	Melastomataceae		Herb	Whole plant	May and June	Pork	Clear *heat*, improve digestion, treat hepatitis, treat enteritis	HKSP-25
*Thlaspi arvense* L.	Brassicaceae	[kʰea^53^tsa^55^]	Herb	Leaf	Summer and fall	Pig bag, pork intestine	Clear* heat*, improve digestion, detoxify, treat diabetes	HKSP-05
*Alternanthera sessilis* (L.) R.Br. ex DC.	Amaranthaceae	[poɡ^24^fo^55^tsɿ^53^], [ma^53^tʰin^55^sɑŋ^55^]	Herb	Root	Whole year	Pork, pork ribs	Clear* heat*, improve digestion, treat hepatitis, treat toothache	HKSP-14
*Amaranthus spinosus* L.	Amaranthaceae	[læ^53^ɕiŋ^241^], [io^55^lə^53^ɕiŋ^241^]	Herb	Root	Spring, summer and fall	Pork ribs, pork intestine	Clear* heat*, diminish inflammation, treat cholecystitis, treat cholelithiasis	HKSP-06
*Portulaca oleracea* L.	Portulacaceae	[mo^55^tsʰɿ^53^xae^53^]	Herb	Whole plant	Spring and summer	Pork, pork ribs	Clear* heat*, induce diuresis, detoxify, diminish inflammation	HKSP-08
*Lysimachia clethroides* Duby	Primulaceae		Herb	Whole plant	Fall	Pork and pork liver	Nourish women, induce diuresis	HKSP-26
*Hedyotis diffusa* Willd.	Rubiaceae	[pʰoɡ^24^fo^55^sɛ^24^sɛ^24^tsʰau^53^]	Herb	Whole plant	Summer and fall	Chicken	Clear* heat*, induce diuresis, prevent and treat cancer, diminish inflammation	HKSP-12
*Paederia scandens* (Lour.) Merr.	Rubiaceae	[ɡie^53^pʰi^24^tʰɛn^11^]	Liana	Root	Summer and fall	Pork ribs, fish	Clear* heat*, induce diuresis, postpartum recovery	HKSP-27
*Lycium chinense* Mill.	Solanaceae	[ɡie^53^tɕiɡ^53^]	Shrub	Leaf	Summer and fall	Mutton, dog meat	Nourishing, tonify kidney and liver	HKSP-32
*Leonurus artemisia* (Lour.) S.Y.Hu	Lamiaceae	[i^24^mea^55^tsʰau^53^]	Herb	Whole plant	Spring and summer	Old hen	Relieve cough, treat cold, treat gynecopathy, postpartum recovery	HKSP-04
*Prunella vulgaris* L.	Lamiaceae	[hɔ^53^kʰeat^55^sʰau^53^]	Herb	Whole plant	July to August	Pork	Clear *heat*, improve eyesight, treat mumps, treat toothache	HKSP-03
*Rabdosia serra* (Maxim.) H.Hara	Lamiaceae	[tʰea^53^vuŋ^11^liŋ^11^]	Herb	Whole plant	Summer and fall	Fish	Clear *heat*, treat hepatitis, treat cholecystitis	HKSP-20
*Scutellaria barbata* D. Don	Lamiaceae	[pɑn^241^tɕi^55^liŋ^11^]	Herb	Whole plant	Summer and fall	Pork ribs, rabbit	Diminish inflammation, relieve swelling and pain, treat traumatic injury	HKSP-10
*Artemisia argyi* H.Lév. & Vaniot	Asteraceae	[ŋai^241^tsʰau^53^]	Herb	Whole plant	May to August	Chicken	Clear *heat*, treat rheumatism, diminish inflammation, stop cough	HKSP-09
*Elephantopus scaber* L.	Asteraceae	[tʰi^55^dɑn^31^tʰiɛ^11^]	Herb	Root	Summer and fall	Duck	Clear *heat*, detoxify, stop cough, induce diuresis and reduce edema	HKSP-21
*Gnaphalium affine* D. Don	Asteraceae	[poɡ^24^tʰiɛ^11^ɡɑŋ^55^]	Herb	Whole plant	Spring	Pork ribs, rabbit	Stop cough, treat tracheitis and bronchitis, treat rheumatism	HKSP-15
*Inula cappa* (Buch.-Ham. ex D. Don) DC.	Asteraceae	[vu^55^tʃuo^53^ɡai^55^]	Herb	Root	Summer and fall	Pork	Dispel wind and relieve pain, detoxify, treat rheumatism	HKSP-23
*Laggera alata* Nanth.	Asteraceae	[yŋ^24^eŋ^53^saŋ^55^tiŋ^55^]	Herb	Whole plant	Summer and fall	Rabbit	Clear *heat*, diminish inflammation, treat cold, stop cough, treat rheumatism	HKSP-24
*Taraxacum mongolicum* Hand.-Mazz.	Asteraceae	[pɑu^214^pɑu^214^tæŋ^55^]	Herb	Whole plant	Whole year	Pork ribs	Clear* heat*, detoxify, induce diuresis and reduce edema, treat hepatitis	HKSP-11
*Eryngium foetidum* L.	Apiaceae	[tsʰɿ^24^tɕʰiɑŋ^11^]	Herb	Stem and leaf	Whole year	Pig bone	Tonify stomach, treat *cold*, diminish inflammation	HKSP-22

The order of species is followed by APG 3

### Quantitative analysis

Quantitative analysis was performed to evaluate the plant composition of medicinal soups and to seek how important and closed of each plant species to the local livelihood, healthcare, and daily diets. The taxonomic status, parts used, and categories of medicinal uses were calculated and analyzed [31]. In order to evaluate the importance of each species to the local community, two indices were used, namely, the cultural food significance index (CFSI) and the relative frequency of citation (RFC).

The CFSI was used to evaluate the cultural significance of an edible species [32, 33]. Specifically, the CFSI was calculated on the basis of the following formula: CFSI = QI × AI × FUI × PUI × MFFI × TSAI × FMRI × 10^−2^, where QI is the frequency of quotation (mention) by participants, AI is the availability of a species, FUI is the frequency of utilization, PUI is the parts used index, MFFI is multi-functional food use, TSAI is the taste score of the plant, and FMRI is the food-medicinal role score [32]. The relative frequency of citation was calculated to show the local importance of each species [33, 34] using the following formula: RFC = FC/*N*, where FC is the number of informants who cited the use of a particular species, and *N* is the total number of informants [34]. Mastery of traditional ethnobotanical knowledge for soup making was evaluated based on the average number of plant species that informants listed.

## Results and discussion

### The analysis of plants used in medicinal soups

A total of 42 plant species from 25 botanical families and 41 genera were identified in this study as being used for making medicinal soups by Hakka informants at the study sites in West Fujian (Table 1). All 42 plant species were angiosperms. Asteraceae was the most prevalent family among the surveyed plants with 6 species represented, while Lamiaceae and Fabaceae were the second most prevalent families, with 4 species each. Almost every other genus contained only 1 plant species except *Ficus*, which contained two species: *Ficus gasparriniana* var. *laceratifola* and *Ficus hirta*.

**Fig. 2:**
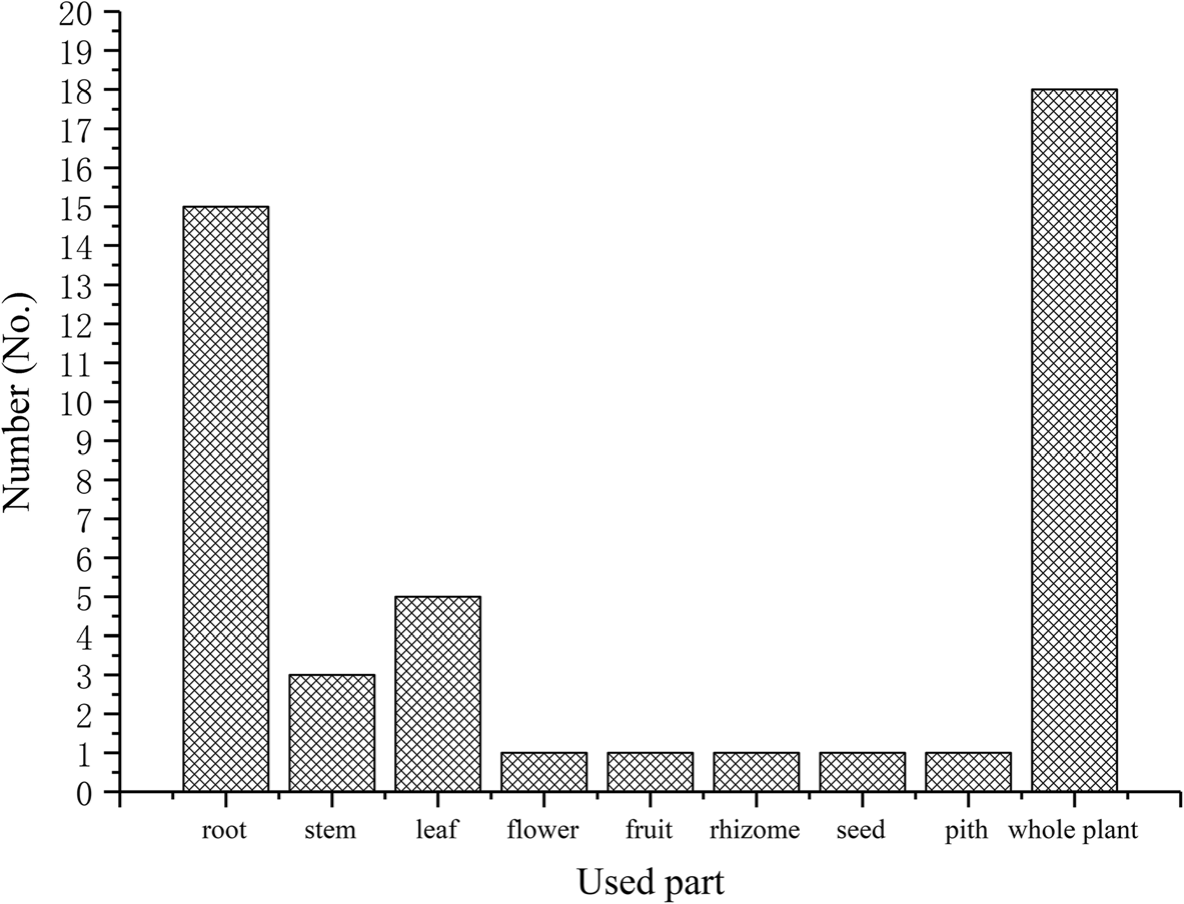
Statistical analysis of the parts of the plants used in medicinal soups

All of the soup-making plants can be purchased from local markets and most of them are collected from the wild except for the seeds of *Nelumbo nucifera* which are extensively cultivated. There were four types of plant habit represented among the surveyed botanicals for making medicinal soups. Twenty-six species (62.0%) were herbs, 13 species (31.0%) were shrubs, two species were lianas (namely *Pueraria lobata* and *Paederia scandens*), and one species was tree (*Morus alba*). While the local Hakka refers to all medicinal plants as “Yao Gen,” which means “medicinal roots” in Mandarin, not all medicinal plants used for soups actually use the plant root. Specifically, the Hakka informants use nine parts of the plants for making medicinal soups, including the root, stem, leaf, flower, fruit, seed, rhizome, stem pith, and whole plant. However, based on our collected data (Fig. 2), the root or the entire plant was used most frequently to make medicinal soups, with 15 species (35.7%) being used for their roots and 18 species (42.9%) for the entire plants. For several species, multiple parts of the plant are used for making medicinal soups including the following: *Prunella vulgaris*, *Tadehagi triquetrum*, *Osbeckia chinensis*, *Morus alba*, and *Rosa laevigata*.

More than half of informants reported that the time of year when specific plants were harvested varied on the basis of the availability and medicinal properties of specific plant parts. The roots, rhizomes, and stems were reported to be usually harvested during the fall and winter because this is the time that they have the greatest potency of the medicinal properties. For example, the rootstalk of *Smilax glabra* and the stem of *Ricinus communis* are regarded to be the most medicinal during the winter. Accordingly, the leaf or the whole plant was usually collected before the flowering phase or in the strongest growth period. Many Hakka reported that the specific medicinal plants collected for making soups varied by season changed according to local ethnomedicine and ethnonutrition systems: people become *overheated* in the summer while they need to dispel *coldness* and receive extra nourishment in the fall and winter. For example, *Artemisia argyi*, which is used by Hakka for clearing *heat* in the human body, is collected in the summer, while *Lysimachia clethroides*, which is used for nourishing, is collected in the fall. The concepts of *heat* (*Yang-heat*) syndrome and *cold* (*Yin-cold*) syndrome here are similar to the philosophy of traditional Chinese medicine, which in Western terms would be an imbalance of homeostasis [35]. The *heat* does not just mean fever or feverishness. It also includes any flushed face, thirst, irritability and restlessness, constipation, deep-colored urine, reddened tongue, and rapid pulse. The *cold* can refer to pallor, intolerance of cold, absence of thirst, loose stools, clear profuse urine, pale tongue, and *slow* pulse, not just bodily cold [36]. The stem of *Ricinus communis* (wild castor) is collected on the Winter Solstice as the Hakka believe that the medicinal potency of the stem is at its peak on this day. All informants reported that once medicinal plants are harvested, they are first dried and then stored, regardless of the time of harvest. This way, dried medicinal plants are available for use in soup making and other uses throughout the year.

**Fig. 3:**
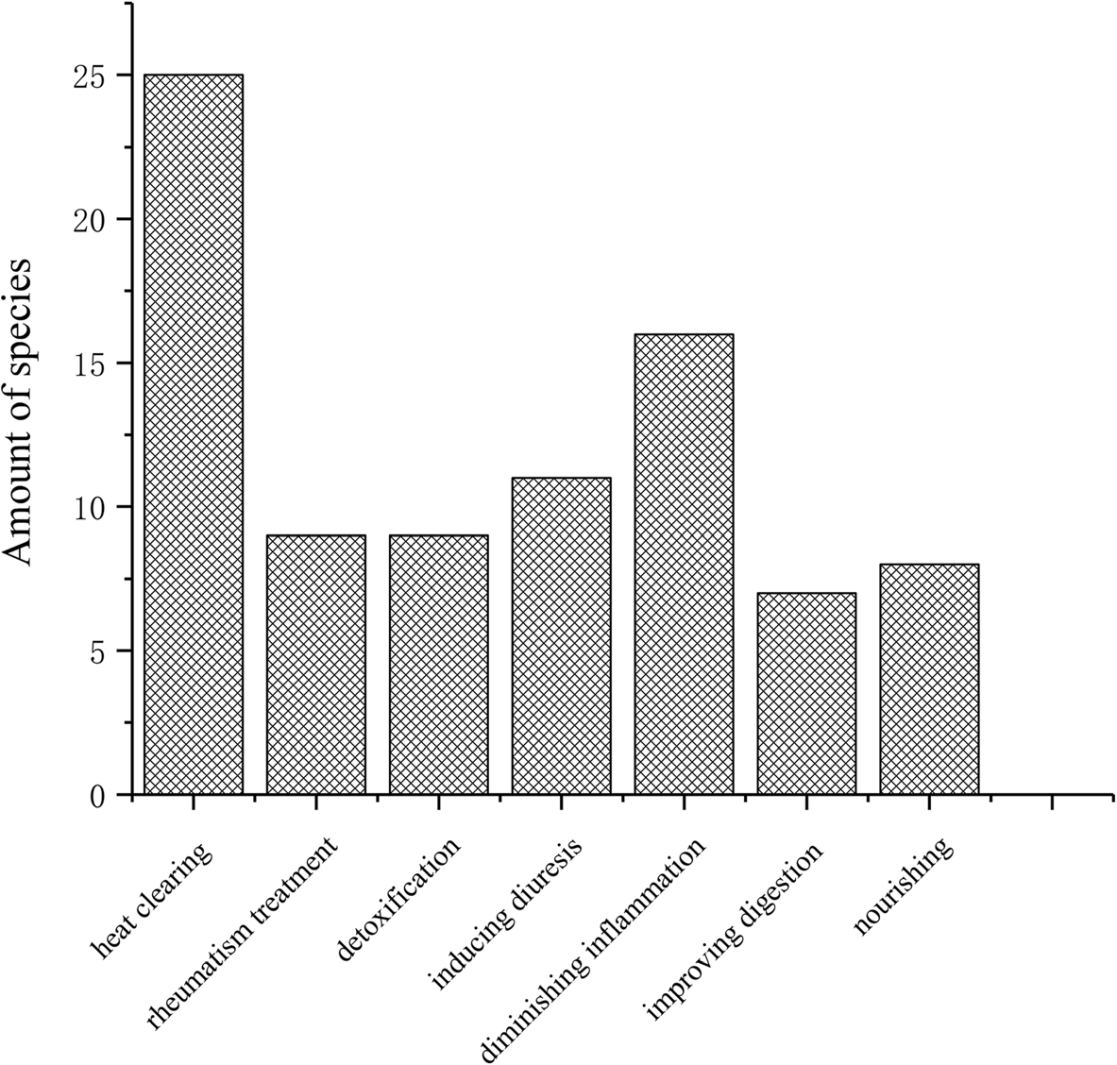
Analysis of the medicinal effects of plants used in soups

The specific medicinal uses of surveyed plants are depicted in Fig. 3. Findings demonstrate that the Hakka informants pay close attention to the balance of *heat* and *cold* in the body. More than half the species (59.5%) were reported for clearing inner *heat* in the human body while 19.0% of species are used to provide nourishment to counteract *cold*. In addition, a notable percentage of the documented plants (38.0%) are used for their anti-inflammatory properties including to treat hepatitis, cholecystitis, tracheitis, and bronchitis. Nine species are used for treating rheumatism by local communities, and 11 species are used for inducing diuresis. The plants for treating rheumatism and inducing diuresis can help local Hakka to better deal with their humid environment and strenuous physical labor. In addition, nine of the documented plant species were used as anti-venoms. Anti-venoms are helpful remedies in the forested montane parts of West Fujian that provides habitat to many poisonous snakes and insects. A total of 7 plant species were used to support digestion, which is particularly helpful for traditional diets that lack refines foods. A few species were used for treating toothache, preventing or treating diabetes, or aiding in postpartum recovery. Several species had multiple reported medicinal uses. For example, the stem pith of *Juncus effusus* was consumed for clearing inner *heat* and diuretic effects. Anecdotal evidence suggests that this plant was historically used as a lamp wick.

### The cultural food significance index and relative frequency of citation of plants used to make medicinal soups

The cultural food significance index (CFSI) of the documented species ranged from 4.7 to 3981.3 with a mean and standard deviation of 508.4 and 840.4. The relative frequency of citation (RFC) of the documented species ranged from 0 to 1 with a mean and standard deviation of 0.55 and 0.30. The rank of all soup-making plants by comparing CFSI is listed in Table 2. The species with higher CFSI are considered more important in traditional diets by local Hakka informants. Three species had both the highest CFSI and RFC due to their high popularity, abundance, and potent medicinal effects including *Houttuynia cordata*, *Laggera alata*, and *Anoectochilus roxburghii*. During our study, some informants reported that they stopped consuming *Houttuynia cordata* because of the information on social media platforms claiming that this species may cause kidney and liver cancer. Particularly, the compound aristolactam, a derivative of aristolochene, has been detected in *Houttuynia cordata* [37]. However, there is no clinical evidence to date that suggests that the consumption of *Houttuynia cordata* is carcinogenic in humans. *Hedyotis diffusa*, which also had both high CFSI and RFC values, is believed by Hakka informants to possess very high medicinal value. *Hedyotis diffusa* is also a popular medicinal plant in other places of South China with a long history of use [38]. *Artemisia argyi* and *Gnaphalium affine* had relatively high CFSI values because they had high multi-functional values. In addition to being used in medicinal soups, *Artemisia argyi* and *Gnaphalium affine* are also used by Hakka in the preparation of *Ban*, a local dish made mainly from rice powder that is traditionally consumed for ceremonies such as on Tomb-Sweeping Day. *Leonurus artemisia* and *Ricinus communis* had a high relative frequency of citation values but were not highly ranked by their CFSI values. The higher RFC of *Leonurus artemisia* may be attributed to their use by Hakka women during postpartum recovery. Additionally, the collection and consumption of *Ricinus communis* on Winter Solstice is a special local custom which leads to the higher RFC value of this species.

**Table 2:** Comparison on cultural food significance index (CFSI) of traditional Hakka soup-making plants in West Fujian

Plant name	AI	FUI	PUI	MFFI	TSAI	FMRI	QI	RFC	CFSI	Rank of CFSI
*Houttuynia cordata*	4.0	4.8	3.0	1.0	9.0	4.8	160	1.00	3981.3	1
*Laggera alata*	3.1	3.9	3.0	1.0	9.0	4.8	160	1.00	2507.0	2
*Anoectochilus roxburghii*	4.0	2.5	3.0	1.0	9.0	5.0	160	1.00	2160.0	3
*Artemisia argyi*	3.9	3.0	3.0	2.5	7.5	4.7	66	0.41	2041.5	4
*Gnaphalium affine*	3.8	2.3	3.0	2.5	7.5	4.4	77	0.48	1665.6	5
*Hedyotis diffusa*	3.1	2.3	3.0	1.0	7.5	4.7	160	1.00	1206.4	6
*Scutellaria barbata*	1.9	3.5	3.0	1.0	9.0	4.0	145	0.91	1041.4	7
*Nelumbo nucifera*	4.0	4.7	1.0	1.0	9.0	3.2	160	1.00	866.3	8
*Prunella vulgaris*	2.0	2.2	3.0	1.0	7.5	4.8	156	0.98	737.1	9
*Thlaspi arvense*	3.3	2.7	3.0	1.0	6.5	3.6	115	0.72	712.2	10
*Portulaca oleracea*	3.3	3.1	1.5	1.0	6.5	4.7	148	0.93	693.8	11
*Juncus effusus*	3.3	4.1	1.0	1.0	7.5	4.1	152	0.95	632.4	12
*Leonurus artemisia*	2.0	1.9	3.0	1.0	6.5	4.6	160	1.00	545.4	13
*Ficus hirta*	3.0	2.5	1.5	1.0	9.0	3.4	134	0.84	468.5	14
*Lycium chinense*	3.7	4.2	1.5	1.0	6.5	4.1	56	0.35	347.9	15
*Ricinus communis*	1.9	2.7	1.0	1.0	7.5	3.9	152	0.95	228.1	16
*Ficus gasparriniana* var. *laceratifola*	1.8	2.3	1.5	1.0	9.0	3.0	122	0.76	206.4	17
*Rhodomyrtus tomentosa*	2.1	2.9	1.5	1.0	6.5	3.0	99	0.62	176.6	18
*Hemerocallis citrina*	3.3	2.4	0.8	1.0	9.0	2.0	122	0.76	130.4	19
*Rabdosia serra*	0.8	2.3	3.0	1.0	6.5	3.8	82	0.51	113.6	20
*Pholidota chinensis*	0.9	1.6	3.0	1.0	6.5	3.8	106	0.66	112.2	21
*Abrus cantoniensis*	0.6	2.8	3.0	1.0	6.5	3.8	78	0.49	98.4	22
*Paederia scandens*	1.7	2.5	1.5	1.0	5.5	3.1	79	0.49	88.2	23
*Phymatopteris hastata*	2.1	1.5	3.0	1.0	7.5	3.5	25	0.16	61.8	24
*Rosa laevigata*	1.3	2.2	1.5	1.0	6.5	3.0	69	0.43	57.7	25
*Litsea cubeba*	1.9	1.3	1.5	1.0	6.5	4.3	50	0.31	51.0	26
*Osbeckia chinensis*	0.6	2.0	3.0	1.0	6.5	3.1	62	0.39	44.2	27
*Uraria crinita*	0.7	1.9	3.0	1.0	5.5	3.3	59	0.37	42.7	28
*Morus alba*	2.4	1.7	1.5	1.0	6.5	3.3	33	0.21	42.3	29
*Inula cappa*	1.0	1.7	1.5	1.0	7.5	3.8	56	0.35	40.2	30
*Gynostemma pentaphyllum*	2.0	1.9	2.0	1.0	6.5	3.2	24	0.15	37.9	31
*Lysimachia clethroides*	1.0	0.9	3.0	1.0	6.5	3.0	70	0.44	36.9	32
*Alternanthera sessilis*	1.1	1.5	1.5	1.0	6.5	3.4	54	0.34	29.3	33
*Tadehagi triquetrum*	1.2	1.7	1.5	1.0	6.5	2.0	67	0.42	26.7	34
*Smilax glabra*	1.0	1.7	1.5	1.0	6.5	3.3	47	0.29	25.6	35
*Elephantopus scaber*	1.3	1.7	1.5	1.0	6.5	2.9	38	0.24	23.0	36
*Amaranthus spinosus*	1.0	1.5	1.5	1.0	6.5	3.2	42	0.26	20.3	37
*Taraxacum mongolicum*	1.0	1.5	1.5	1.0	6.5	3.8	26	0.16	14.3	38
*Pueraria lobata*	0.7	1.5	1.5	1.0	5.5	3.6	38	0.24	12.0	39
*Berchemia lineata*	1.1	1.2	1.5	1.0	6.5	3.0	27	0.17	10.0	40
*Polygala fallax*	0.5	1.0	1.5	1.0	6.5	3.3	62	0.39	10.0	41
*Eryngium foetidum*	1.0	0.5	2.0	1.0	5.5	3.9	22	0.14	4.7	42

AI is the availability of a species; FUI is the frequency of utilization; PUI is the parts used index; MFFI is multi-functional food use; TSAI is the taste score of the plant; FMRI is the food-medicinal role score; QI is the frequency of quotation (mention) by participants; RFC is the relative frequency of citation

### Special cases of plants used in medicinal soups

Two collected species that were reported to be poisonous, *Ricinus communis* and *Hemerocallis citrina*, are also consumed by Hakka informants in medicinal soups (Table 1). Castor bean (*Ricinus communis*) is usually cultivated for the production of valuable oil used in industry and medicinal fields [39]. Castor bean seeds can be highly toxic due to their ricin and ricinine content [40]. The castor plant is used to provide nourishment in soups and tonics, particularly in the winter, and its harvest and use in Hakka communities coincide with its harvest time. Further toxicological studies are necessary to determine the toxicity of the castor plant and how its edibility is affected by when it is harvested, what parts of the plant are ingested, and how it is prepared.

The fresh flower of *Hemerocallis citrina* contains colchicine, a poisonous compound [41]. The Hakka thus have careful preparation methods for this flower. Before cooking the flowers of *Hemerocallis citrina*, local Hakka typically remove the stamen of the flowers and then blanch them in boiling water. The plants are then washed and soaked in cold water for at least 20 min before they are cooked in soups. The soup of *Hemerocallis citrina* flower has a slightly sour taste and has high nutritional and medicinal value. It is typically consumed by the Hakka in the summer.

A soup made from *Monascus ruber* (red rice yeast) has medicinal properties that are of particular benefit to women. The soup is made from old capon, red rice yeast powder, ginger, and Hakka rice wine in a concoction with a red color and a pungent odor. Soup made from *Monascus ruber* is associated with local health claims in Hakka communities by all informants to effectively aid in postpartum recovery and dispel *coldness* in women. *Monascus ruber* is an important fungus in traditional Chinese medicine, as it has been reported to have many beneficial properties, including anti-tumor, cholesterol-reducing, and blood pressure-reducing properties [42, 43]. In addition to its use in medicinal soups, red rice yeast is also used to make traditional rice bread and to brew red rice wine in Hakka communities.

### Pairing rules for plants used in medicinal soups

Over time, the Hakka have become well-versed in pairing different plants and ingredients in order to create soups with specific medicinal attributes as well as to enhance the flavor of soups. According to Hakka informants, medicinal soups vary in their medicinal attributes and flavors based on the specific plants and other ingredients, including the pairing of ingredients. When choosing soup-making ingredients and how to pair these ingredients, Hakka informants reported that they take into consideration the medicinal properties of plants as well as other attributes such as flavor and if the plant is *hot* or *cold* according to their ethnonutrition system. The Hakka ethnonutrition system is based on a similar system as traditional Chinese medicine of viewing different plants and foods as possessing specific characteristics of *heat* and *cold*. *Cold* foods alleviate *heat* syndrome and are usually consumed for reducing and clearing internal *heat* or fever. Conversely, *warm* and *hot* foods can help to dispel *coldness* and are commonly used to promote appetite and provide nourishment. This belief of *cold* and *hot* foods is used as a guiding principle when making medicinal soups. In addition, choosing medicinal plants and ingredients that complement each other or that work in synergy to promote good health is another key principle of the Hakka ethnonutrition system. Another guiding principle for creating soups draws from the Doctrine of Signatures where certain visceral animal organs are selected to support tonifying attributes to human visceral organs based on the same functions. For example, a soup made from the stem of *Juncus effusus* L. coupled with pork heart is considered to discharge fire in the human heart. Soup made from dry fruit of *Rosa laevigata* Michx and pork kidney is used for arresting kidney problems. Additionally, a soup made from leaves of *Thlaspi arvense* L. and pork intestine is used for intestinal detoxification and to promote digestion.

This food-matching principle fully embodies the style and features of TCM, which originated from the ancient central plains of China and may have been adopted by the Hakka people during their historical migration. Additionally, we also found that Hakka cuisine has similar characteristics to the cuisine of the She socio-linguistic group that are indigenous to West Fujian Province [44]. Both groups are known to assign significant value to the *hot* and *cold* nature of foods and incorporate different foods into their diet to prevent or treat diseases [45].

Findings suggest that the basis of food pairings of the Hakka ethnonutrition system is an amalgamation of local knowledge coupled with that drawn from traditional Chinese medicine and aboriginal She people. This knowledge likely developed and was modified during the migration of the Hakka, which included changes in the natural resources of their new environment [46]. Previous research suggests that the diversity of this knowledge may increase or decrease depending on spatiotemporal factors [47]. Due to the isolated geography of Southwestern China, coupled with the integration of inherent local food knowledge of the Hakka traditional food system, knowledge of medicinal plants held by the Hakka has evolved and diversified over time.

### Traditional ecological knowledge regarding medicinal plants used for soups

**Fig. 4:**
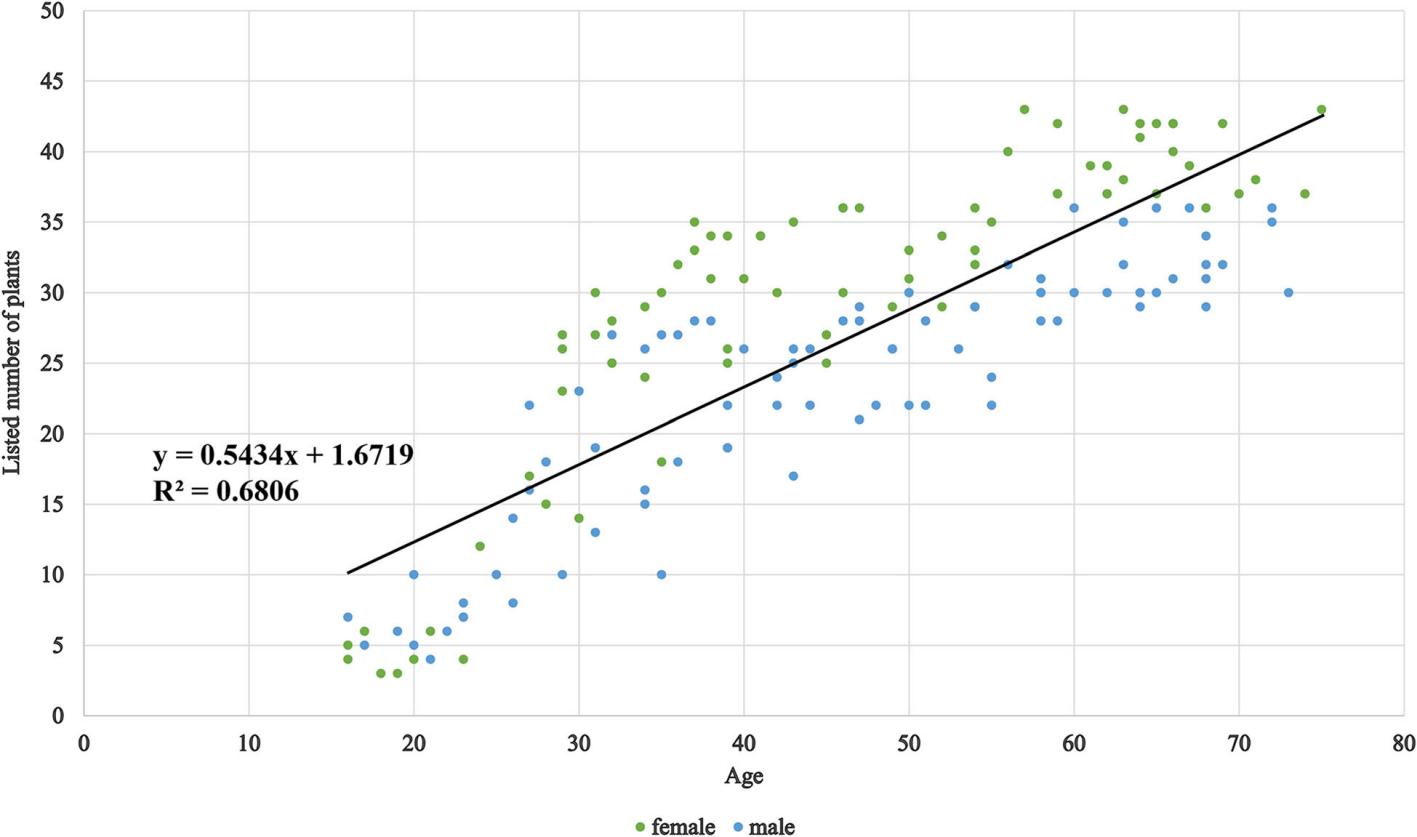
Number of species listed based on the age and gender of the Hakka people. The green dots stand for female informants and the blue dots stand for male informant. The regression line is drawn based on informants’ ages and amounts of listed plants

Findings demonstrated that traditional ecological knowledge regarding plants used for making soups varied on the basis of the gender of participants, but not significantly varied in age. Figure 4 is a scattered diagram based on the listed number, genders, and ages of informants. Each dot stands for an informant. Although the regression line seems to show that older people have more knowledge about soup-making plants, the *R*^2^ is only 0.5434, which means that this trend is not significant. The regression analysis was tested based on the participants’ age and the listed numbers. *P* -value is 0.2423, and much higher than 0.05, which indicated that age and listed numbers are not significantly related. On the basis of gender, the one-way ANOVA demonstrated that the amounts of plants listed by local women and men are significantly different, in which the *P* -value is only 0.00013, and much lower than 0.05. According to Fig. 4, most of the women over the age of 30 knew significantly more botanical species for soup making than men in the same age group. The greater knowledge of plants for soup making by women compared with men reflects the distinct traditional gender roles of the Hakka people, where women are responsible for preparing meals. Of all informants, herb vendors, and healers demonstrated the greatest knowledge of plants used to make medicinal soups; these informants could identify at least 35 botanical species for soup making during interviews. As depicted in Fig. 4, Young informants under the age of 25 years could only distinguish a few species, including those that were most common and had high RFC values. Most of the young informants were not very good at local Hakka languages and could only speak the vernacular names of the plants they usually consumed, yet the last generation was still good at Hakka language and familiar to the plant’s Hakka names. The generation gap on language might be the reason causing the younger generation could not identify the plants they used. Also, local people like using dry plants for storage and making soup, which looks dramatically different from the fresh ones and increasing the difficulty for the young generation to inherited the related knowledge. Additionally, all of the older females (> 55 years old) were able to list almost all species recorded in this study.

Even though the analysis suggested that the age and the plant knowledge holding are not significantly related, the data still showed that the younger generation is not very knowledgeable about these plants. With the rapid development and urbanization of Hakka communities, residents have migrated into larger towns and cities to seek a better standard of living and modern amenities. Consequently, knowledge and skills of being able to identify and collect medicinal plants for soup making are gradually being lost by younger Hakka. The Hakka language, which is spoken and not written, is also becoming extinct as the younger generation is learning Mandarin in schools rather than their traditional language. It has long been recognized that ecological knowledge is embedded in language, and the loss of language contributes to the loss of local traditional knowledge [48]. Furthermore, land-use change is resulting in a loss of plant species, which will further result in the loss of traditional ecological knowledge and traditional food systems of consuming wild medicinal and edible plants. Efforts are thus needed to protect local biodiversity, traditional ecological knowledge, and the practice of preparing and consuming medicinal soups as part of sustainable diets in Hakka communities.

### Commercial availability of plants used in medicinal soups in West Fujian

We evaluated the commercial availability of the plants used to make medicinal soups in markets of West Fujian by asking the price of wild medicinal plants and the degree of difficulty for obtaining them from the wild. The exploitation and utilization ratio of these resources were relatively low. Almost all plants for medicinal soup were collected in the wild. Herb vendors would regularly go to the countryside to buy medicinal herbs collected in the wild by villagers, and then resell them at a profit in urban areas. Most plants were sold fresh or dried, or in simple packaging. Selling wild plants do not notably contribute to local livelihoods based on household income.

Local biodiversity is also threatened by harvesting for commercial purposes. Wild plants are often overharvested because some local collectors are not aware of plant sustainable harvesting practices. The most representative case of this is the orchid species *Anoectochilus roxburghii*, which is called the “golden herb” by local people and is listed in the China Red Data Book. It is one of the most valuable ornamental, edible, and medicinal herbs used for liver protection and treating hypertension and diabetes [49, 50]. *A. roxburghii* is scarce in the wild and is now mostly cultivated for resale by herb vendors. Market analysis has shown that the price of dried wild *A. roxburghii* is high and can be up to 10,000 yuan/kg, while the cultivated plant retails for 2000 yuan/kg (1 yuan = 0.1456 USD). It is quite challenging to grow *A. roxburghii* because of its low germination rate, *slow* growth, and requirement of a high-altitude growth environment [49, 51]. Therefore, the demand for *A. roxburghii* significantly exceeds the supply, resulting in local government development of the *A. roxburghii* seed industry.

However, efforts of the government and local companies to protect and sustain the development of medicinal plants have not been adequate. Enhanced cooperation is needed between governmental and scientific entities and local communities to ensure the preservation and sustainable development of wild medicinal plants that support traditional food systems and associated well-being.

## Conclusion

Medicinal soups prepared using a diversity of plants are an integral part of the traditional food systems and local concepts of ethnonutrition of Hakka communities in West Fujian. Medicinal soups reflect Hakka ethnonutrition concepts of disease prevention and treatment. By conducting ethnobotanical investigations, we identified 42 plant species (25 families and 41 genera) that are used to make medicinal soups by Hakka informants. The taxonomic distribution, plant type, edible parts, medicinal effects, and time of harvest varied and showed regional characteristics. In addition, the way in which different plants were paired with other ingredients in medicinal soups followed principles of traditional Chinese medicine and local practices. The quantitative analysis highlighted the cultural importance of specific medicinal herbs and their associated knowledge. However, knowledge of medicinal plants is at risk of being lost by the Hakka due to the depletion of local biodiversity and urbanization of the study site communities. Therefore, integrated efforts are required between the local communities and government to ensure the preservation of vital ethnobotanical knowledge that supports environmental and human well-being.

## Data Availability

All data generated or analyzed during this study are included in this published article and its supplementary information files.
